# 2-(2-Methoxy­phen­yl)butane­dinitrile

**DOI:** 10.1107/S1600536810013462

**Published:** 2010-04-17

**Authors:** Xiang-Zi Li, Zhi-Jun Feng, Yan Yu, Wei-Li Shen, Yin Ye

**Affiliations:** a College of Chemistry and Materials Science, Anhui Key Laboratory of Functional Molecular Solids, Anhui Normal University, Wuhu 241000, People’s Republic of China; b Department of Chemistry, WanNan Medical College, Wuhu 241000, People’s Republic of China

## Abstract

In the title compound, C_11_H_10_N_2_O, the butane­dinitrile unit adopts a synclinal conformation. The crystal packing is stabilized by weak inter­molecular C—H⋯N hydrogen bonding.

## Related literature

The title compound is an important inter­mediate in drugs synthesis, see: Obniska *et al.* (2005 ▶). For the synthesis, see: Johnson *et al.* (1962 ▶).
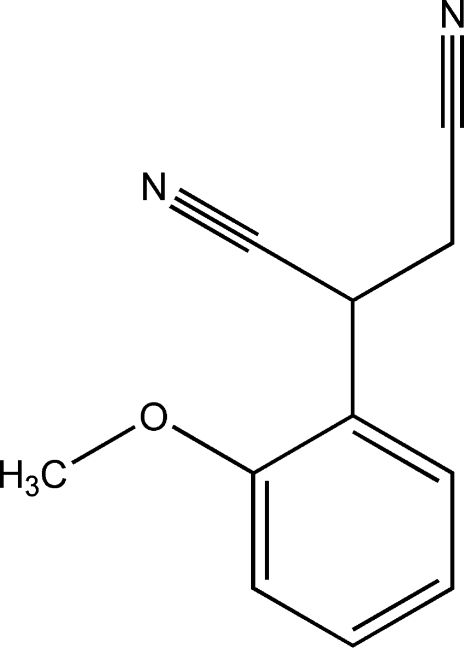


## Experimental

### 

#### Crystal data

C_11_H_10_N_2_O
*M* *_r_* = 186.21
Monoclinic, 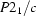

*a* = 12.393 (9) Å
*b* = 5.405 (4) Å
*c* = 15.216 (10) Å
β = 102.947 (8)°
*V* = 993.3 (12) Å^3^
*Z* = 4
Mo *K*α radiation
μ = 0.08 mm^−1^
*T* = 298 K
0.37 × 0.25 × 0.14 mm

#### Data collection

Bruker SMART APEXII CCD area-detector diffractometer
Absorption correction: multi-scan (*SADABS*; Sheldrick, 1996 ▶) *T*_min_ = 0.970, *T*_max_ = 0.985
7820 measured reflections
2292 independent reflections
1549 reflections with *I* > 2σ(*I*)
*R*_int_ = 0.026

#### Refinement

*R*[*F* ^2^ > 2σ(*F* ^2^)] = 0.042
*wR*(*F* ^2^) = 0.116
*S* = 1.04
2292 reflections
128 parameters
H-atom parameters constrained
Δρ_max_ = 0.12 e Å^−3^
Δρ_min_ = −0.14 e Å^−3^

### 

Data collection: *SMART* (Bruker, 2000 ▶); cell refinement: *SAINT* (Bruker, 2000 ▶); data reduction: *SAINT*; program(s) used to solve structure: *SHELXTL* (Sheldrick, 2008 ▶); program(s) used to refine structure: *SHELXTL*; molecular graphics: *SHELXTL*; software used to prepare material for publication: *SHELXTL*.

## Supplementary Material

Crystal structure: contains datablocks I, global. DOI: 10.1107/S1600536810013462/xu2746sup1.cif

Structure factors: contains datablocks I. DOI: 10.1107/S1600536810013462/xu2746Isup2.hkl

Additional supplementary materials:  crystallographic information; 3D view; checkCIF report

## Figures and Tables

**Table 1:** Hydrogen-bond geometry (Å, °)

*D*—H⋯*A*	*D*—H	H⋯*A*	*D*⋯*A*	*D*—H⋯*A*
C3—H3⋯N2^i^	0.93	2.50	3.404 (3)	165
C8—H8⋯N2^ii^	0.98	2.50	3.262 (3)	135

Symmetry codes: (i) 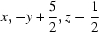; (ii) 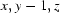.

**Fig. 1.**
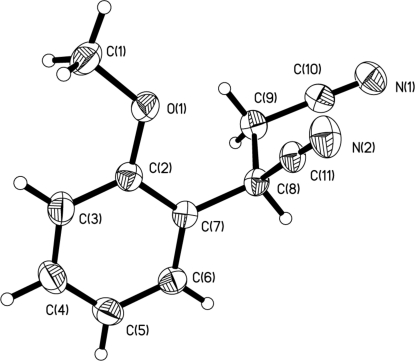
The molecular structure of (I), showing the atom-labelling scheme. Displacement ellipsoids are drawn at the 30% probability level.

**Fig. 2.**
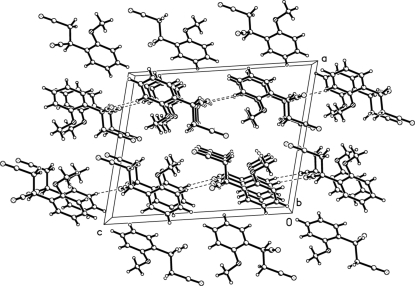
A packing diagram of (I) viewed down the b axis. Dotted lines show the hydrogen bonds.

## supplementary crystallographic information

### Comment

The title compound is an important intermediate in drugs synthesis (Obniska *et al.*, 2005). In this paper, we report the structure of the title compound (I). In (I), the succinonitrite moiety adopts a cis conformation. Two cyanide groups, (N1—C10 and N2—C11), are not coplane. However, the methoxy group is almost coplanar with the the mean plane of the phenyl (C2/C3/C4/C5/C6/C7). The crystal packing is stabilized by two intermolecular non-classic C—H···N hydrogen bonds.

### Experimental

The compound (I) was obtained by reaction of (*Z*)-ethyl-2-cyano-3-(2-methoxyphenyl)acrylate and NaCN in ethanol-water mixture according to the reported method (Johnson *et al.*, 1962). Single crystals suitable for X-ray diffraction were obtained by evaporation of an ethanol solution at room temperature.

### Refinement

H atoms were placed in calculated positions and refined using a riding model with C—H = 0.93-0.98 Å. U_iso_(H) = 1.5U_eq_(C) for methyl and 1.2U_eq_(C) for the others.

### Crystal data

**Table:**

C_11_H_10_N_2_O	*F*(000) = 392
*M**_r_* = 186.21	*D*_x_ = 1.245 Mg m^−^^3^
Monoclinic, *P*2_1_/*c*	Mo *K*α radiation, λ = 0.71073 Å
Hall symbol: -P 2ybc	Cell parameters from 2518 reflections
*a* = 12.393 (9) Å	θ = 2.8–26.1°
*b* = 5.405 (4) Å	µ = 0.08 mm^−^^1^
*c* = 15.216 (10) Å	*T* = 298 K
β = 102.947 (8)°	Block, colorless
*V* = 993.3 (12) Å^3^	0.37 × 0.25 × 0.14 mm
*Z* = 4	

### Data collection

**Table:**

Bruker SMART APEXII CCD area-detector diffractometer	2292 independent reflections
Radiation source: fine-focus sealed tube	1549 reflections with *I* > 2σ(*I*)
graphite	*R*_int_ = 0.026
φ and ω scans	θ_max_ = 27.7°, θ_min_ = 1.7°
Absorption correction: multi-scan (*SADABS*; Sheldrick, 1996)	*h* = −16→16
*T*_min_ = 0.970, *T*_max_ = 0.985	*k* = −6→6
7820 measured reflections	*l* = −18→19

### Refinement

**Table:**

Refinement on *F*^2^	Primary atom site location: structure-invariant direct methods
Least-squares matrix: full	Secondary atom site location: difference Fourier map
*R*[*F*^2^ > 2σ(*F*^2^)] = 0.042	Hydrogen site location: inferred from neighbouring sites
*wR*(*F*^2^) = 0.116	H-atom parameters constrained
*S* = 1.04	*w* = 1/[σ^2^(*F*_o_^2^) + (0.051*P*)^2^ + 0.1131*P*] where *P* = (*F*_o_^2^ + 2*F*_c_^2^)/3
2292 reflections	(Δ/σ)_max_ < 0.001
128 parameters	Δρ_max_ = 0.12 e Å^−^^3^
0 restraints	Δρ_min_ = −0.14 e Å^−^^3^

### Special details

**Table:**

Geometry. All esds (except the esd in the dihedral angle between two l.s. planes) are estimated using the full covariance matrix. The cell esds are taken into account individually in the estimation of esds in distances, angles and torsion angles; correlations between esds in cell parameters are only used when they are defined by crystal symmetry. An approximate (isotropic) treatment of cell esds is used for estimating esds involving l.s. planes.
Refinement. Refinement of *F*^2^ against ALL reflections. The weighted *R*-factor wR and goodness of fit *S* are based on *F*^2^, conventional *R*-factors *R* are based on *F*, with *F* set to zero for negative *F*^2^. The threshold expression of *F*^2^ > σ(*F*^2^) is used only for calculating *R*-factors(gt) etc. and is not relevant to the choice of reflections for refinement. *R*-factors based on *F*^2^ are statistically about twice as large as those based on *F*, and *R*- factors based on ALL data will be even larger.

### Fractional atomic coordinates and isotropic or equivalent isotropic displacement parameters (Å^2^)

**Table:**

	*x*	*y*	*z*	*U*_iso_*/*U*_eq_	
C1	0.34900 (15)	1.2727 (3)	0.69677 (11)	0.0644 (5)	
H1A	0.3731	1.1717	0.6529	0.097*	
H1B	0.4106	1.3657	0.7303	0.097*	
H1C	0.2924	1.3843	0.6667	0.097*	
N1	0.45207 (12)	0.7505 (3)	1.07568 (10)	0.0728 (4)	
O1	0.30584 (9)	1.11902 (19)	0.75662 (6)	0.0581 (3)	
C2	0.22452 (11)	0.9545 (3)	0.72065 (9)	0.0461 (3)	
N2	0.22425 (13)	1.2438 (2)	0.95010 (9)	0.0660 (4)	
C3	0.17425 (13)	0.9391 (3)	0.63011 (10)	0.0585 (4)	
H3	0.1945	1.0466	0.5889	0.070*	
C4	0.09351 (14)	0.7621 (3)	0.60142 (11)	0.0642 (5)	
H4	0.0591	0.7523	0.5406	0.077*	
C5	0.06344 (13)	0.6016 (3)	0.66096 (11)	0.0605 (4)	
H5	0.0102	0.4810	0.6406	0.073*	
C6	0.11252 (11)	0.6197 (3)	0.75120 (10)	0.0500 (4)	
H6	0.0913	0.5117	0.7919	0.060*	
C7	0.19287 (11)	0.7958 (2)	0.78245 (8)	0.0409 (3)	
C8	0.24600 (11)	0.8102 (2)	0.88203 (9)	0.0422 (3)	
H8	0.2064	0.6939	0.9129	0.051*	
C9	0.36818 (12)	0.7315 (3)	0.90375 (10)	0.0518 (4)	
H9A	0.3741	0.5645	0.8819	0.062*	
H9B	0.4096	0.8402	0.8727	0.062*	
C10	0.41624 (12)	0.7404 (3)	1.00039 (11)	0.0547 (4)	
C11	0.23445 (11)	1.0568 (3)	0.91933 (9)	0.0457 (3)	

### Atomic displacement parameters (Å^2^)

**Table:**

	*U*^11^	*U*^22^	*U*^33^	*U*^12^	*U*^13^	*U*^23^
C1	0.0803 (11)	0.0570 (10)	0.0640 (10)	−0.0141 (8)	0.0332 (9)	0.0021 (8)
N1	0.0724 (10)	0.0769 (10)	0.0604 (9)	0.0051 (7)	−0.0034 (7)	0.0056 (7)
O1	0.0726 (7)	0.0561 (6)	0.0471 (6)	−0.0181 (5)	0.0167 (5)	0.0032 (5)
C2	0.0515 (8)	0.0429 (8)	0.0447 (7)	0.0002 (6)	0.0129 (6)	−0.0007 (6)
N2	0.0978 (11)	0.0466 (8)	0.0543 (8)	0.0086 (7)	0.0183 (7)	−0.0036 (6)
C3	0.0678 (10)	0.0649 (10)	0.0435 (8)	−0.0002 (8)	0.0138 (7)	0.0049 (7)
C4	0.0639 (10)	0.0787 (12)	0.0451 (8)	0.0010 (9)	0.0018 (7)	−0.0066 (8)
C5	0.0527 (9)	0.0664 (10)	0.0593 (9)	−0.0081 (7)	0.0057 (7)	−0.0090 (8)
C6	0.0500 (8)	0.0463 (8)	0.0543 (9)	−0.0010 (6)	0.0131 (6)	0.0000 (6)
C7	0.0450 (7)	0.0372 (7)	0.0413 (7)	0.0039 (5)	0.0112 (6)	0.0004 (5)
C8	0.0507 (8)	0.0357 (7)	0.0413 (7)	0.0008 (6)	0.0124 (6)	0.0033 (5)
C9	0.0544 (8)	0.0499 (9)	0.0495 (8)	0.0090 (6)	0.0081 (6)	0.0022 (6)
C10	0.0530 (8)	0.0493 (9)	0.0579 (9)	0.0065 (7)	0.0041 (7)	0.0054 (7)
C11	0.0555 (8)	0.0434 (8)	0.0382 (7)	0.0030 (6)	0.0102 (6)	0.0039 (6)

### Geometric parameters (Å, °)

**Table:**

C1—O1	1.4225 (18)	C4—H4	0.9300
C1—H1A	0.9600	C5—C6	1.375 (2)
C1—H1B	0.9600	C5—H5	0.9300
C1—H1C	0.9600	C6—C7	1.383 (2)
N1—C10	1.133 (2)	C6—H6	0.9300
O1—C2	1.3632 (17)	C7—C8	1.513 (2)
C2—C3	1.381 (2)	C8—C11	1.468 (2)
C2—C7	1.3928 (19)	C8—C9	1.536 (2)
N2—C11	1.1327 (18)	C8—H8	0.9800
C3—C4	1.382 (2)	C9—C10	1.458 (2)
C3—H3	0.9300	C9—H9A	0.9700
C4—C5	1.365 (2)	C9—H9B	0.9700
			
O1—C1—H1A	109.5	C5—C6—H6	119.5
O1—C1—H1B	109.5	C7—C6—H6	119.5
H1A—C1—H1B	109.5	C6—C7—C2	118.83 (13)
O1—C1—H1C	109.5	C6—C7—C8	119.89 (12)
H1A—C1—H1C	109.5	C2—C7—C8	121.28 (12)
H1B—C1—H1C	109.5	C11—C8—C7	112.07 (10)
C2—O1—C1	118.29 (12)	C11—C8—C9	110.19 (11)
O1—C2—C3	124.58 (13)	C7—C8—C9	112.84 (11)
O1—C2—C7	115.18 (12)	C11—C8—H8	107.1
C3—C2—C7	120.24 (13)	C7—C8—H8	107.1
C2—C3—C4	119.32 (14)	C9—C8—H8	107.1
C2—C3—H3	120.3	C10—C9—C8	111.60 (12)
C4—C3—H3	120.3	C10—C9—H9A	109.3
C5—C4—C3	121.07 (15)	C8—C9—H9A	109.3
C5—C4—H4	119.5	C10—C9—H9B	109.3
C3—C4—H4	119.5	C8—C9—H9B	109.3
C4—C5—C6	119.48 (15)	H9A—C9—H9B	108.0
C4—C5—H5	120.3	N1—C10—C9	178.67 (17)
C6—C5—H5	120.3	N2—C11—C8	177.91 (15)
C5—C6—C7	121.04 (14)		
			
C1—O1—C2—C3	−5.4 (2)	C3—C2—C7—C6	1.5 (2)
C1—O1—C2—C7	174.73 (13)	O1—C2—C7—C8	0.32 (18)
O1—C2—C3—C4	179.19 (15)	C3—C2—C7—C8	−179.53 (12)
C7—C2—C3—C4	−1.0 (2)	C6—C7—C8—C11	−123.69 (14)
C2—C3—C4—C5	−0.5 (2)	C2—C7—C8—C11	57.39 (17)
C3—C4—C5—C6	1.4 (3)	C6—C7—C8—C9	111.21 (15)
C4—C5—C6—C7	−0.8 (2)	C2—C7—C8—C9	−67.71 (16)
C5—C6—C7—C2	−0.6 (2)	C11—C8—C9—C10	55.55 (15)
C5—C6—C7—C8	−179.58 (13)	C7—C8—C9—C10	−178.33 (11)
O1—C2—C7—C6	−178.61 (12)		

### Hydrogen-bond geometry (Å, °)

**Table:**

*D*—H···*A*	*D*—H	H···*A*	*D*···*A*	*D*—H···*A*
C3—H3···N2^i^	0.93	2.50	3.404 (3)	165
C8—H8···N2^ii^	0.98	2.50	3.262 (3)	135

Symmetry codes: (i) *x*, −*y*+5/2, *z*−1/2; (ii) *x*, *y*−1, *z*.
